# Supplementary material for: Site-selective mapping of metastable states using electron-beam induced luminescence microscopy
Source: Sci Rep. 2020 Sep 24;10:15650. doi: 10.1038/s41598-020-72334-7 (PMC7518257; doi:10.1038/s41598-020-72334-7)
Supplement: Supplementary file 1 — Supplementary file1 [file 41598_2020_72334_MOESM1_ESM.pdf]

Site-selective mapping of metastable states using electron-beam induced luminescence microscopy

R. Kumar[1,*], L.I.D.J. Martin[2], D. Poelman[2], D. Vandenberghe[3], J.De Grave[3], M. Kook[1], M. Jain[1]

[1]Department of Physics, Technical University of Denmark, DTU Risø Campus, Denmark-4000

[2]Department of Solid-state Sciences, University of Ghent, Belgium-9000

[3]Department of Geology, University of Ghent, Belgium-9000

Corresponding author: Raju Kumar (mail id: rajcelos.isp65@gmail.com)

Figure SI 1

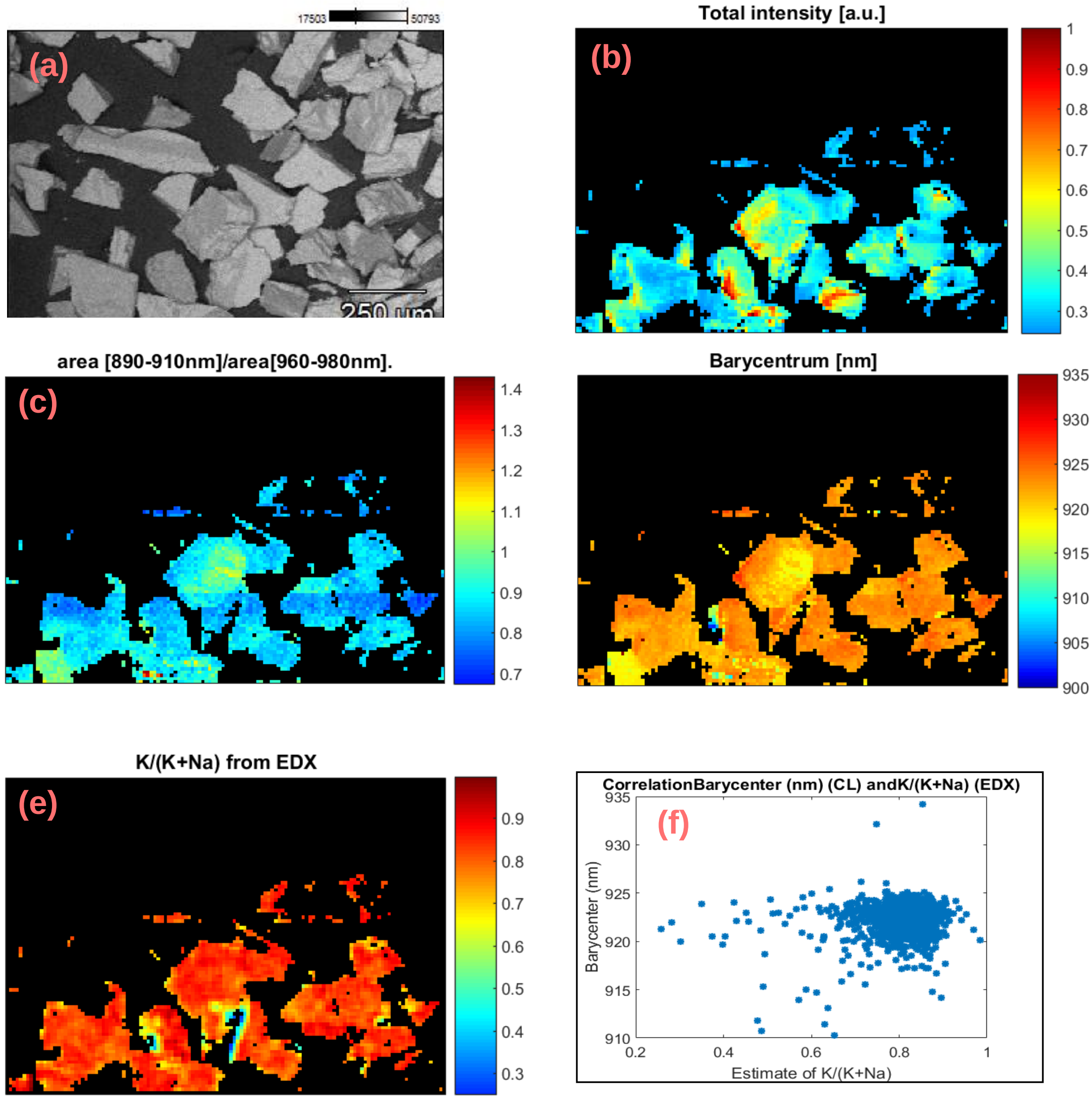

Figure SI 1: CL and elemental data for sample R50. (a) BSE image, (b) cathodoluminescence intensity map; intensity below a value 0.235 I_{max} has been filtered out using a mask, (c) intensity ratio map between the two NIR bands (890-910 nm and 960-980 nm), (d) barycenter map in the range 910-935 nm, (e) relative concentration map of the K content (K/K+Na), and (f) correlation between the IRCL emission barycenter and relative K concentration. These images were generated using Matlab (Version: R2016, Url: https://www.mathworks.com/products/matlab.html)

Figure SI 2

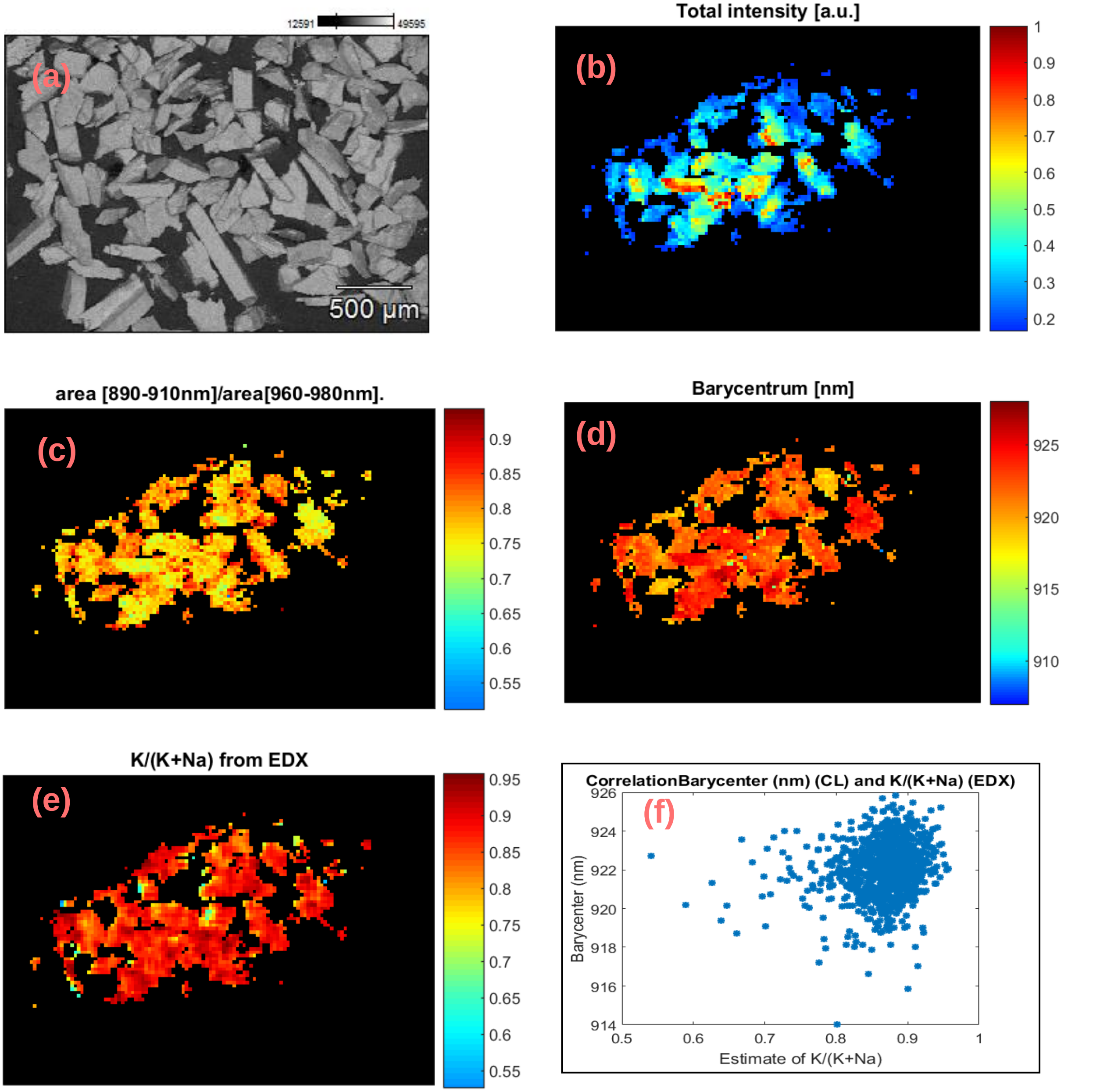

Figure SI 2: CL and elemental data for sample K13. (a) BSE image, (b) cathodoluminescence intensity map; intensity below a value 0.235 I_{max} has been filtered out using a mask, (c) intensity ratio map between the two NIR bands (890-910 nm and 960-980 nm), (d) barycenter map in the range 910-935 nm, (e) relative concentration map of the K content (K/K+Na), and (f) correlation between the IRCL emission barycenter and relative K concentration. These images were generated using Matlab (Version: R2016, Url: https://www.mathworks.com/products/matlab.html)
